# Capgras delusion in postpartum psychosis: a case report

**DOI:** 10.1186/s12991-021-00342-6

**Published:** 2021-03-20

**Authors:** Sulochana Joshi, Mankaji Thapa, Anusha Manandhar, Rabi Shakya

**Affiliations:** Department of Psychiatry, Patan Academy of Health Sciences, Lagankhel, Lalitpur, Nepal

**Keywords:** Capgras, Delusional misidentification syndrome, Phenomenon, Postpartum

## Abstract

**Background:**

Capgras delusion is one of the delusional misidentification syndromes characterized by the belief by the patient that the close person is replaced by an imposter who looks physically the same. It rarely occurs in Postpartum Psychosis. An intriguing phenomenon with ongoing debates, particularly about its feature and prevalence, its course, occurrence, and phenomenon in the postpartum period are poorly understood.

**Case presentation:**

A 26-year-old Nepalese woman presented to the emergency for abnormal behavior on her 9th postpartum day. Capgras delusion was observed for 2 days during her hospital stay. Other psychotic symptoms appeared progressively and were treated as a case of Postpartum Psychosis.

**Conclusion:**

This case describes the temporal sequence of various psychopathologies during Postpartum Psychosis including Capgras delusion. We attempt to explain the occurrence of Capgras delusion in Postpartum Psychosis.

## Background

Capgras delusion is a belief by a patient that a double exists who is physically, yet not psychologically, identical to him/herself and/or to his/her closed ones. It occurs in clear consciousness [1–3] and is associated with various neurological and psychiatric conditions [2, 3]. It is considered a nonspecific delusion rather than a distinct disorder [4]. It is encompassed in the syndrome of delusional misidentification along with other delusional misidentifications like Fregoli syndrome, Subjective doubles, Inter-metamorphosis [3, 5]. It is a negative misidentification with defensive maneuver and ambivalent emotions of love-hate and paranoid interpretation [6, 7]. It is the most common delusion among the misidentification syndromes but a rare syndrome in itself [2, 8].

During the postpartum period, the most common psychiatric illness is postpartum depression, followed by postpartum psychosis and postpartum blues [9]. Capgras delusion is not common in the puerperium period [10, 11]. Postpartum psychosis, a serious psychiatric condition with a prevalence of one to two per 1000 childbirth in the general population, is 100 times more common in women with bipolar disorder or a history of postpartum psychosis [12, 13]. The clinical features include odd affect and mood incongruent unusual psychotic symptoms related to a child’s altered identity, persecution, and changeling [9]. It is generally considered an episode of bipolar disorder with accompanying psychotic symptoms [9, 14, 15]. In this case report, we describe the occurrence of Capgras delusion in Postpartum Psychosis.

## Case presentation

A 26-year-old housewife from a rural mid-western part of Nepal presented to the emergency on her 9th postpartum day. She had undergone Caesarean Section for a transverse lie. Her husband complained of her talking out of context, frequent change of clothes, and pulling off her clothes as well as fisting her hands for the last 6 days. She had had similar symptoms 15 months back when she lost her 6 month pregnancy. With Olanzapine 10 mg, she had been stable but she discontinued her medication 6 months back after she discovered her pregnancy. She had no history of substance use or chronic illnesses including diabetes mellitus or hypertension. She had a well-adjusted premorbid personality.

She was doing well until her 3rd postpartum day when she learned that her baby had developed jaundice. She became restless and started talking out of context saying ‘the witch will take away her baby’. She frequently changed her clothes, cried, and threw away her own and the baby’s clothes. She used to pull her ornaments, self-mutter, fist her hands, and stare at the family members. Such behavior continued till the 9th postpartum day when the patient was brought to the emergency. On her mental state examination, she was irritable and uninterested, had increased psychomotor activity, hallucinatory behavior, and persecutory idea without insight. Baseline investigations (Complete Blood Count, Renal Function Test, Urine Routine, and Microscopic Examination and Thyroid Function Test) were normal. The Brief Psychiatric Rating Scale (BPRS) score was 48 (Table 1) and Clinical Global Impressions-Severity(CGI-S) score was 6 (Table 2) then.

**Table 1:** Brief Psychiatric Rating Scale (BPRS) scores

Day of admission	2	4	6	11	17	27
BPRS score	48	57	50	42	28	19

**Table 2:** Clinical Global Impressions-Severity (CGI-S) scores

Day of admission	2	4	8	11	17	27
CGI-S score	6	6	6	5	5	2

From day 3 (12th postpartum day) in the psychiatry ward, she frequently expressed that her husband and the baby would be taken away by the ‘witch’. After a few days, she started quarreling with her husband claiming that he was, instead, an imposter of her husband. She was firm on her belief and became irritable when confronted with. She did not elaborate much except claiming that she ‘knew’ he was not her husband. She would not talk to her husband and would become angry when approached.

On day 4, she also expressed that ‘evil spirit’ talked in her ears saying they have come to take her away which made her fearful and self-muttering. Her mental state examination showed irritable affect, auditory hallucination, persecutory delusion, and Capgras delusion.

From day 5, she began recognizing her husband but expressed that his clothes were her enemies. She claimed that he was trying to harm her, had brought her here to ‘sell’ her. She further expressed that he had been cheating on her. She continued to express persecutory, referential delusion, and auditory hallucinations until day 19 when she gradually became shakeable on her belief. After day 19, her persecutory belief involved around the neighbors and towards the females only claiming the neighbors to be the evil spirits trying to harm her.

From day 27, she became almost premorbid with partial insight and without signs and symptoms of hallucination and suspiciousness. After maintaining well for 1 week, she was discharged on Sodium Valproate 500 mg twice a day and Olanzapine 20 mg once a day at bedtime. Olanzapine was started on the first day of hospitalization with 5 mg which was gradually optimized to 20 mg with Clonazepam 0.5 mg as the adjunct. Similarly, Sodium Valproate was started after day 17 when she had not improved with 20 mg of Olanzapine. The Clinical Global Impressions-Improvement(CGI-I) scores show gradual improvement with time and medications (Table 3).

**Table 3:** Clinical Global Impressions-Improvement (CGI-I) scores

Day of admission	1	8	17	27
CGI-I score	7	5	2	1

## Conclusion and discussion

In this case report, we describe the case of Capgras delusion in the postpartum period from the evolution of delusion until its resolution along with other associated symptoms in temporal sequence, which is the strength of the case report. To our knowledge, only two cases of Capgras delusion with psychotic features have been reported during the postpartum period, highlighting that delusion in postpartum psychosis is rare [16, 17]. Its true prevalence remains unknown. A systematic review of published studies between 1923 and 2016 has found 255 case reports with Capgras delusion [11]. In our case, the 2 days (in the hospital stay) of Capgras delusion later transformed into persecutory delusion along with the development of auditory hallucination and referential delusion which remained for a longer duration (24 days). This case report elaborates on the course of Postpartum psychosis with a brief duration (2 day in-hospital stay) of Capgras delusion which might, in part, explain its rarity. It is possible to be missed if it is present only for a few days as in our case or when the patient has not been under the regular supervision of an expert.

On one hand, Capgras delusion is believed to be amorphous. On the other hand, postpartum psychosis is considered to be an overt presentation of bipolar disorder with symptoms including various types from affective, psychotic to catatonic [9, 11–15]. As postpartum psychosis is characterized by varied symptoms especially delusions and hallucination and affective symptoms, this Capgras delusion could be the initial symptom among many other symptoms of Postpartum Psychosis as in our case [9, 13, 15, 18, 19]. Hence, the case report attempts to suggest that Capgras delusion could be the initiating phenomenon in Postpartum Psychosis.

Thus, we have attempted to explain that Capgras delusion can be present, though rare, in Postpartum Psychosis.

### Consent

Written informed consent was obtained from the patient’s caretaker for publication of this case report. A copy of the written consent is available for review by the Editor-in-Chief of this journal.

## Data Availability

Not applicable.
